# Completion of three-dose hepatitis B vaccination cycle and associated factors among health care workers in the Greater Accra Region of Ghana

**DOI:** 10.1371/journal.pone.0298771

**Published:** 2024-04-16

**Authors:** Vivian Efua Senoo-Dogbey, Francis Anto, Reginald Quansah, Anthony Danso-Appiah

**Affiliations:** 1 Department of Public Health, School of Nursing and Midwifery, University of Ghana, Legon, Accra, Ghana; 2 Department of Epidemiology, School of Public Health, University of Ghana, Legon, Accra, Ghana; 3 Department of Behavioural, Environmental and Occupational Health, University of Ghana, Legon, Accra, Ghana; University for Development Studies, GHANA

## Abstract

**Background:**

Despite the availability of a safe and effective vaccine coupled with the awareness of the potential risk of Healthcare Workers acquiring Hepatitis B Virus infection, some HCWs never get vaccinated. Generally, hepatitis B vaccination coverage globally is below the expected level as adherence has remained poor in various healthcare settings, especially in developing countries. The objective of this study was to assess the completion of a three-dose Hepatitis B virus vaccination cycle and associated factors among healthcare workers in the Greater Accra Region of Ghana.

**Methods and materials:**

An analytical cross-sectional study was conducted and included 363 healthcare workers selected using probability sampling procedures. The participants were recruited from five facilities within the Greater Accra Region in the first half of 2018. A pretested questionnaire was used to collect data which was analyzed using SPSS version 21. The proportion of healthcare workers receiving the recommended 3 doses of the hepatitis vaccine was computed. The multivariable analysis procedure identified the factors associated with adherence to the receipt of three doses of the hepatitis B vaccine. Odds ratios were estimated with corresponding confidence intervals with the level of significance set at 0.05.

**Results:**

A total of 340 sample units were included in the analysis. Most of the participants (252/340, 74.1%) were females, mainly nurses/midwives (162/340, 47.6%) with a mean age of 34.5 (SD ±7.7). A high proportion of the participants (82.7%) have tertiary/post-tertiary level education and ever participated in at least one training workshop on the prevention of blood-borne infections (80.6%). Overall vaccination uptake was 60.9% (207/340) (95% CI = 55.7%-66.1%). Complete vaccination coverage (three doses) was 46.8% (159/340). High-risk perception (AOR = 4.0; 95% CI = 1.3–12.5), and previous training in infection prevention (AOR = 2.8; 95% CI = 1.1–7.5) were significantly associated with adherence to receipt of three doses of hepatitis B vaccine.

**Conclusion:**

Adherence to three-dose hepatitis B vaccination cycles is not universal among the healthcare workers in the Greater Accra Region. Receipt of the three-dose regimen is significantly associated with high-risk perception and attendance of training in infectious disease prevention. Interventions to increase risk perception and training in the prevention of blood-borne infections could improve adherence to complete/full vaccination protocol among healthcare workers who are at constant risk of exposure to the hepatitis B virus.

## Background

The Hepatitis B Virus (HBV) is a DNA virus belonging to the *Hepadnaviridae* family of viruses. This virus is responsible for a potentially life-threatening liver infection called HBV infection. Globally, 296–350 million people are currently at the chronic stage of the infection with 1.5 million new infections every year. In 2019 alone, HBV infection accounted for close to 820, 000 deaths globally, resulting from hepatocellular carcinoma and liver cirrhosis which are its major complications [1]. The burden of this infection is high in sub-Saharan Africa and Asia, where close to 70–95% of the population have serological evidence of exposure to the virus [2,3] an indication of marked disparity in the burden and progress towards elimination across the world. In Ghana, estimates show 12–14% of infections among the general population [4,5].

HBV is a blood-borne infection that is effectively transmitted through exposure to contaminated blood and body fluids. Among the 60 or more disease-causing organisms responsible for blood-borne transmissible infectious diseases, HBV is one of the most frequently transmitted pathogens to Healthcare Workers (HCWs). This characteristic of the virus is the basis for its recognition as an important occupational hazard for HCWs all over the world [6]. The global pooled prevalence of HBsAg which indicates current HBV infection among HCWs was currently estimated to be 2.3% [7]. Among HCWs, in Ghana, the burden could be higher with an estimated prevalence of 5.9% [8].

Hepatitis B virus infection is a vaccine-preventable infection. A safe and effective vaccine has been available since 1982. Reports are that 90–95% of immunocompetent individuals who vaccinate before the age of 40 years have the potential to develop immunity against the virus and stand lifelong protection [9]. Despite the availability of a safe and effective vaccine coupled with the awareness of the potential risk of HCWs acquiring HBV infection, some HCWs never get vaccinated. Therefore, HBV vaccination coverage globally is still below the expected level as adherence has remained poor in various healthcare settings, especially in developing countries [10]. HBV vaccination coverage has been reported to be as low as 4.5% in Rwanda, 5.4% in Tanzania, and 14.2% in Nigeria [11–13].

Not all persons who get vaccinated against HBV attain complete vaccination status [14]. Complete vaccination is receipt of all three doses of the vaccine at the recommended schedule, and this is considered optimal for seroprotection against HBV. The rate of Anti-HBs positivity is also known to be dose-dependent [15]. Anti-HBs positivity has been reported to increase gradually from 35% after the first dose to 90% or more after 3^rd^ dose hence the importance of the three-dose regimen especially among individuals who are at high risk of HBV infection [15].

In Ghana, there is a paucity of information regarding the protection of HCWs against HBV; with reports of only 53.4% of HCWs in the Greater Accra Region receiving at least one dose of the HBV vaccine [16] and 44.4% of nurses in the Tamale metropolis also receiving at least one dose [17]. Evidence from these two studies suggests that HBV vaccination coverage is not optimum among Ghanaian HCWs which could be the reason for the high HBV prevalence in this population. Secondly, failure to receive the recommended doses of the HBV vaccine could also affect the level of seroprotection among this population and therefore render HCWs susceptible to HBV infection. In Ghana, even though few studies estimated HBV vaccination coverage among HCWs [16,17] none of these studies specifically assessed the adherence to receipt of a complete cycle of three doses of HBV vaccine and associated factors. Against this background, this study was undertaken to assess the completion of three-dose Hepatitis B virus vaccination cycle and associated factors among health care workers in the Greater Accra Region of Ghana.

## Methods and materials

### Study area

This study was carried out in the Greater Accra Region of Ghana. The region is one of sixteen administrative regions in the country. There are 29 health administrative districts and 1297 health institutions consisting of one regional hospital, 10 district hospitals, 118 general hospitals, 21 polyclinics, 292 clinics, 35 health centers, 707 CHPS compounds, and 106 maternity homes. The rest are three university hospitals, two teaching hospitals, and two psychiatric hospitals. This region is the most populous in Ghana followed by the Ashanti Region but has the smallest land area with the average number of HCWs being 10,055 [18]. Greater Accra Region is located in the south-central part of the country, bordering the Central Region to the west, the Volta Region to the east, the Eastern Region to the north, and the Gulf of Guinea to the south [19]. The study was undertaken in the first half of 2018.

### Study design

This study was a facility-based analytical cross-sectional study which was conducted in the Greater Accra Region of Ghana.

### Study population and inclusion criteria

The study was carried out among healthcare workers made up of doctors, laboratory professionals hospital sanitation workers (orderlies), anaesthetists, physician assistants, nurses, and midwives. These HCWs were recruited as study participants on the basis that they have direct contact with the blood and body fluids of patients and therefore are at high risk of occupational exposure to HBV and other blood-borne infections. We recruited only participants who had been working in their current facility for six months or more since facility-level factors are known to influence HBV vaccination practice. Also, only HCWs who gave informed written consent were allowed to be part of the study.

### Sample size estimation

Using the Cochran formula, n = (Z^2^pq)/d^2^ [20] where: n = sample size, Z = the z-score that corresponds with a 95% confidence interval (1.96), p = estimated proportion of health workers who received a complete cycle of three doses of HBV vaccine (31.1%, = 0.311) [21] q = estimated proportion of health workers who received less than the complete cycle of three doses of the HBV vaccine (1–0.311 = 0.689), d = margin of error set at 5% (0.05). The ‘estimate of 31.1% was obtained from a pilot study done at Prampram Heath center and also supported by a related study done in Nigeria [21]. This was necessary because no known study in Ghana sought to specifically assess adherence to the receipt of the complete cycle of three doses of HBV vaccine and associated factors. By adjusting for 2% non-response [8] as described in a related study, a sample size of 363 was estimated to be adequate for the determination of factors associated with the uptake of three doses of HBV among healthcare workers.

### Sampling procedure

There are five strata or categories of health facilities under the Greater Accra Regional Health Directorate (Reginal hospital, District hospitals, Polyclinics, Health centers, and CHPS compounds). A total of 5 health facilities were selected from each existing stratum. The selection of facilities from each of the five strata was done randomly using simple random sampling procedures (lottery method) except for the regional hospital which was purposively chosen as there is only one regional hospital in the Greater Accra Region. Proportional allocation of the samples was done for each of the five selected facilities to ensure that facilities contribute samples or participants proportionally to the study. The number of respondents per healthcare worker category per facility was also proportional to size. A systematic random sampling procedure was finally employed to select individual respondents from among eligible healthcare worker categories using a predetermined sampling interval for each category. The sampling interval *(k)* was obtained by dividing the total number of healthcare workers in that specific professional category by the allocated number of samples per each category of HCWs or professional category. A random starting point was identified and every K^*th*^ element in the sampling frame was selected. The sampling interval differed from HCW category to category as there are variations in the population sizes of the professional categories. Several revisits were made due to the shift system at the health facilities in order the meet the selected health workers. The majority of the participants were recruited from the Regional Hospital followed by the District Hospital. Nurses and midwives were also in the majority *Table 1.*

**Table 1:** Background characteristics of study participants (N = 340).

	Variable	No.	%
**Age group (years)**			
	21–30	127	37.4
	31–40	153	45.0
	41–50	43	12.6
	51–60	17	5.0
**Sex**			
	Male	88	25.9
	Female	252	74.1
**Category of staff**			
	Doctor	69	20.3
	Nurse/Midwife	162	47.6
	Anaesthetist	15	4.4
	Laboratory Professionals	40	11.8
	Orderly/Sanitation workers	35	10.3
	Physician Assistant^¥^	19	5.6
**Educational level**			
	No formal Education	6	1.8
	Primary	17	5.0
	Secondary	36	10.6
	Tertiary	240	70.6
	Post Tertiary	41	12.1
**Duration of employment** ^**β**^			
	<10 Years	260	76.5
	≥10 Years	80	23.5
**Work unit** *			
	Unit with High Exposure	155	45.6
	Unit with low to moderate exposure	185	54.4
**Facility Type**			
	CHPs compound**	19	5.6
	Health Centre	28	8.2
	Polyclinic	56	16.5
	District Hospital	80	23.5
	Regional Hospital	157	46.2
**Training in infection prevention and control**			
	Received Training	274	80.6
	Not Trained	66	19.4
**Perception of Risk of Infection** ***			
	High	295	86.8
	Low	45	13.2

^*¥*^ *Physician assistants are healthcare workers who are trained as prescribers to cater for ailments as a primary health care initiative in Ghana Duration of employment is based on the median duration of work and also supported by previous study [24]*

*Work units where exposure to contaminated blood and body fluids is very likely under the type of procedures performed on patients

**Community Bases Health Planning Services compound

*** Low and high risk classification based on mean score [22] and previous study [36].

### Data collection and quality control

The study utilized a structured data collection instrument with 30 items with questions adopted from the literature on recommended HBV vaccination practices for HCWs. To assess participants’ perception of the risk of HBV infection, we adapted and used the Champions Health belief model subscale which is a standard subscale for measuring perception of health behaviour [22]. The Main data collection tool was developed specifically for this study and reviewed by experts in the field including officials of the occupational health and safety department of the Ghana Health Service. The research instrument was pretested among 20 HCWs selected from the same professional categories as the main study. The pretesting was done at the Prampram Health Centre which is a government-owned primary level facility in the Greater Accra Region. The Prampram health center was not one of the sampled health facilities for the main study. The pretesting procedure helped to reword some questions to make them clearer and re-arrange some to provide for a more logical flow of the questions. Data collection was done through self-administered interviews which were moderated by research assistants who were graduate nurses selected from facilities other than the five study sites. They had two days of training on skills and procedures for questionnaire administration, complying with sampling procedures, as well as following the consenting processes. The research assistants were also trained on the correct documentation of information from the participants’ vaccination cards. Daily checks were conducted for the completeness of the questionnaire. Data entry was done independently by two individuals and comparisons were made. Frequency distributions were run for all variables to check for omissions or missing data. Duplicates were also carefully examined and removed.

Some operational definitions utilised in this study are outlined as follows: (a) Risk perception for HBV which, defined as the belief of being susceptible to HBV and HBV having serious consequences was classified as low and high based on the mean score values [22,25] (b)Work unit was defined based on the extent of blood and body fluid contact suffered by HCWs. Thus, HCWs who work at OPD, antenatal clinics etc with minimum exposure to blood and body fluids were classified as working in units with low to moderate risk whereas HCWs who work in theatres, wards etc where there is a great possibility of blood and body fluid contact were considered as working in units with higher risk of exposure to HBV [2]. (c) Physician assistants were defined in this study as health professionals with training in the management of health conditions in primary healthcare settings. (d)Adherence to HBV vaccination was defined as completing the three doses of HBV vaccine recommended for all HCWs. (e) Duration of employment was defined as the length of occupational exposure to HBV and was classified as <10 for those who had not attained 10 years since they were employed and >10 years for those who have been employed for over 10 years. This was based on the median years of employment and supported by a previous study [24]. Adherence to the three-dose HBV vaccine was computed using the proportion of HCWs receiving three doses or more of the HBV vaccine.

### Data processing and analysis

Data entry, cleaning, and analysis were done using Statistical Package for Social Scientists (SPSS) version 21 software. Age and duration of employment were classified based on information from related studies and median years of work duration for the study participants respectively [23,24]. Composite risk perception scores were dichotomized into good (≥ 50) and poor risk perception (<50) [25]. Only a few missing at-random data were identified and case deletion or removal strategy was used in contrast to insertions [26]. Results were presented as counts (proportions) for categorical variables and mean with Standard Deviations (SD) for quantitative variables. Odds Ratios (OR) with their 95% Confidence Intervals (CI) served to investigate the influence of various factors on the receipt of three doses of HBV vaccine and were obtained through bivariate or multivariable logistic regression analyses while adjusting for potential confounders. Variables with p-value ≤ 02.5 were included in the multivariable model. A p-value of <0.05 was considered significant. The Hosmer–Lemeshow test was used to test the goodness of fit of the logistic regression models.

### Ethics statement

Ethical approval for the conduct of this study was obtained from the Institutional Review Board of the Noguchi Memorial Institute for Medical Research, University of Ghana (*No*: *NMIMR-IRB-CPN 005/17-18)* and the Ethics Review Committee of the Ghana Health Service *(No*. *GHS-ERC006/08/17)*. Permission was also obtained from the Regional Health Director of the Greater Accra Region *(Letter no*. *GHS/GARHD/006/17)* and all the facility heads. Written, informed and signed consent was obtained from each study participant after the nature, purpose, and procedures of the study were thoroughly explained to them. Anonymity was achieved by de-identifying the data by using serial numbers or codes for each participant. Privacy was ensured by saving the research data in a password-protected computer which was accessible to only the principal investigator.

## Results

### Background characteristics of study participants

A total of 363 Health Care Workers (HCWs) participated in the study, with 340 of them providing complete data giving a response rate of 94% therefore 340 sample units were included in the analysis. Most of the participants (252/340, 74.1%) were females, mainly nurses/midwives (162/340, 47.6%) with a mean age of 34.5 (SD ±7.7). A high proportion of the participants (82.7%) have tertiary/post-tertiary level education and ever participated in at least one training workshop on the prevention of blood-borne infections (80.6%). The majority (76.5%) of the participants have less than 10 years of working experience. In all, 45.6% of them work as care providers in units and departments perceived as having a high risk of exposure to blood and body fluids *(Table 1).*

### Uptake of three doses of HBV vaccination (complete vaccination)

Results presented in Table 2 show that, out of the 340 HCWs, 207 (60.9%) received at least one dose of the HBV vaccine of which 159 were adherent to the three-dose regimen (receipt of 3 doses HBV vaccine), giving three (3) doses vaccination adherence of 46.8% among the entire population of HCWs surveyed. However, the three-dose vaccination coverage among the 207 vaccinated HCWs was 76.8% (159/207).

**Table 2:** Uptake of three-dose HBV vaccination (N = 207).

		Three Doses of HBV Vaccine			
Variables	N (207)	n (159)	Per cent (95% CI)	Chi-square	P-value
**Age group (years)**				7.79	0.051
21–30	76	64	84.2(74–91.6)		
31–40	95	67	70.5(60.3–79.4)		
41–50	27	23	85.2(66.3–95.8)		
51–60	9	5	55.6(21.2–86.3)		
**Sex**				0.08	0.779
Female	152	116	76.3(68.7–82.8)		
Male	55	43	78.2(65–88.2)		
**Category of Staff**					0.085
Doctor	44	36	81.8(67.3–91.8)		
Nurse/Midwife	95	68	71.6(61.4–80.4)		
Anaesthetist	11	7	63.6(30.8–89.1)		
Laboratory Professionals	31	29	93.5(78.6–99.2)		
Orderly/sanitation workers	13	9	69.2(38.6–90.9)		
Physician Assistants.	13	10	76.9(46.2–95)		
**Educational level**				***	**0.009**
No formal	4	3	75.00(19.4–99.4)		
Primary	4	0	_		
Secondary	15	13	86.7(59.5–98.3)		
Tertiary	154	122	79.2(72–85.3)		
Post tertiary	30	21	70.0 (50.6–85.3)		
**Perception of risk of infection**				1.95	0.162
Low	23	15	65.2(42.7–83.6)		
High	184	144	78.3(71.6–84)		
**Duration of** **Employment (years)**				1.47	0.225
<10	156	123	78.8(71.6–85)		
≥10	51	36	70.6(56.2–82.5)		
**Training in infection prevention and control**				2.27	0.132
No trained	33	22	66.7(48.2–82)		
Received Training	174	137	78.7(71.9–84.6)		
**Facility Type**					0.185
CHPs Compound	10	8	80(44.4–97.5)	**	
Health center	11	10	90.9(58.7–99.8)		
Polyclinic	35	22	62.9(44.9–78.5)		
District hospital	45	38	84.4(70.5–93.5)		
Regional hospital	106	81	76.4(67.2–84.1)		
**Work Unit**				0.36	0.550
Unit with high exposure	100	75	75.0(65.3–83.1)		
Unit with low to moderate exposure	107	84	78.5(69.5–85.9)		

***Estimated from Fisher’s exact test.

The crude results presented in Table 2 show that the majority of HCWs working in critical units with high exposure rates adhered to the three-dose regimen 78.5% (84/107) compared to 75% (75/100) of those working in units with low to moderate exposure to blood and body fluids. Seventy-eight per cent (144/184) of HCWs with high-risk perception for HBV adhered to the 3-dose regimen. The majority of HCWs, indicating 78.7% (137/174) who received training in blood-borne infection prevention adhered to the three-dose regimen.

### Factors associated with the receipt of three doses of the HBV vaccine

Multivariable logistic regression analysis revealed that participants who perceived themselves as being at high risk for HBV infection had a higher odd of taking all three doses of the vaccine (AOR = 4.0; 95% CI = 1.3–12.5) compared to those who did not see themselves as being at high risk. Similarly, those who have had some training on the prevention of blood-borne infections were about three times more likely to take all three doses compared to those without any training on the prevention of blood-borne infections (AOR = 2.8; 95% CI = 1.0–7.5) (Table 3).

**Table 3:** Logistic regression analysis to determine the association between sociodemographic and occupational factors associated with uptake of ≥ 3 doses of HBV vaccine.

Variable				Crude Estimates		Adjusted Estimates	
		N (207)	n(159)	cOR (95% CI)	p-value	aOR (95% CI)	p-value
**Age group (years)**							
	21–30	76	64	1.0		1.0	
	31–40	95	67	0.4(0.2–1.0)	0.038	0.6(0.2–1.3)	0.18
	41–50	27	23	1.1(0.3–3.7)	0.904	2.8(0.5–15.3)	0.225
	51–60	9	5	0.2(0.1–1.0)	0.050	0.7(0.1–6.3)	0.760
**Sex**							
	Female	152	116	1.0		1.0	
	Male	55	43	1.1(0.5–2.3)	0.779	0.9(0.3–2.2)	0.755
**Category of staff**							
	Doctor	44	36	1.0		1.0	
	Nurse/Midwife	95	68	0.6(0.2–1.4)	0.199	0.5(0.2–1.4)	0.169
	Anesthetist	11	7	0.4(0.1–1.7)	0.201	0.3(0–1.6)	0.146
	Laboratory professionals	31	29	3.2(0.6–16.4)	0.158	3.4(0.5–23.6)	0.219
	Orderly/sanitation workers	13	9	0.5(0.1–2.0)	0.334	0.6(0.1–4.6)	0.586
	Physician Assistant	13	10	0.7(0.2–3.3)	0.695	0.5(0.1–3.2)	0.502
**Education**							
	No formal education	4	3	1.0		1.0	
	Primary	4	0	1.0		1.0	
	Secondary	15	13	2.2(0.1–32.5)	0.576	13.8(0.6–327.5)	0.105
	Tertiary	154	122	1.3(0.1–12.6)	0.838	4.5(0.4–58)	0.246
	Post tertiary	30	21	0.8(0.1–8.5)	0.837	2.1(0.2–30.5)	0.574
**Perception of risk of infection**							
	Low	23	15	1.0		1.0	
	High	184	144	1.9(0.8–4.9)	0.168	4(1.3–12.5)	**0.016**
**Duration of employment (years)**							
	<10	156	123	1.0		1.0	
	≥10	51	36	0.6(0.3–1.3)	0.227	0.5(0.2–1.4)	0.189
**Training in infection prevention and control**							
	Not trained	33	22	1.0		1.0	
	Received Training	174	137	1.9(0.8–4.2)	0.136	2.8(1.1–7.5)	**0.043**
**Facility Type**							
	CHPS compound	10	8	1.0		1.0	
	Health center	11	10	2.5(0.2–32.8)	0.485	1.6(0.1–24.6)	0.755
	Polyclinic	35	22	0.4(0.1–2.3)	0.32	0.3(0–1.9)	0.182
	District hospital	45	38	1.4(0.2–7.8)	0.732	1(0.1–7.3)	0.991
	Regional hospital	106	81	0.8(0.2–4.1)	0.798	0.8(0.1–5.2)	0.846
**Work unit**							
	Unit with high exposure	100	75	1.0		1.0	
	Unit with moderate exposure	107	84	1.2(0.6–2.3)	0.551	0.7(0.3–1.8)	0.496

## Discussion

A facility-based cross-sectional analytical study was conducted among healthcare workers in the Greater Accra Region of Ghana to identify factors associated with the uptake of three or more doses of the hepatitis B vaccine. The study revealed that perceiving oneself as being at high risk of being infected with HBV and receiving in-service training on the prevention of blood-borne infections, increased the odds of taking three doses of the vaccine.

Three doses of the vaccine have been recommended as adequate for protection in adults [27]. Our current study found 46.8% of the study participants taking three doses; thus, less than half of the study population is likely to be protected from future hepatitis B virus infections. A Nigerian study equally documented similar coverage of three doses of HBV vaccine as 48.9% [11]. The coverage reported in this study even though lower than expected, is still higher than most studies done in Africa. Generally, three-dose vaccination coverage is low among HCWs in low-income countries. For example, a systematic review and meta-analysis of studies from 15 African countries (Ghana not included) estimated an overall uptake of three doses of the vaccine to be 24.7%, only 13.4% for Central Africa. Other studies have also reported similarly low levels of uptake of less than 50% among healthcare workers in Ethiopia (25.6%) Nigeria (48.9%) and India (40.0%) [11,28,29].

Failure to comply with the three-dose regimen has serious implications for the safety of healthcare workers since the attainment of seroconversion and for that matter seroprotection after HBV vaccination has been linked with compliance with the three dose-schedule, especially for individuals with delayed seroconversion. In a study by Baghianimoghadam and colleagues, only 59.5% of the participants developed Hepatitis B surface antibodies (anti-HBs) to the required obligatory levels after receiving two doses. Meanwhile, seroconversion and consequent development of anti-HBs to the obligatory levels rose to 99.2% after the uptake of the third dose. Thus, three doses of the vaccine are necessary to increase the seropositivity rate of anti-HBs in adults [30]. Even though some studies have reported comparable efficacy of two HBV vaccine doses [31,32], this may not apply to all population groups, since those particular studies were conducted among children and adolescents. Many other studies support the three-dose regimen as the best for individuals vaccinating as adults, especially HCWs [27].

This current study found high-risk perception for HBV to be significantly correlated with the uptake of three doses of the HBV vaccine. The high-risk perception here refers to a sense of susceptibility or vulnerability to HBV infection. Vaccination against vaccine-preventable infectious diseases is identified as a positive health behavior that is influenced by the level of perceived vulnerability or susceptibility to the health hazard [33]. Reports from a multi-center study that evaluated the influence of risk perception on vaccination also found a strong relationship between high-risk perception and the willingness of HCWs to vaccinate [34]. Similarly, Senoo-Dogbey et al., (2024) in Their study among Ghanaian HCWs found low-risk perception for HBV to be significantly associated with failure to comply with the three-component HBV vaccination recommendations for HCWs [35].

On the other hand, Abiodun et al (2017), in their study among HCWs in Nigeria, reported a low-risk perception for HBV with a corresponding very low HBV vaccination coverage [36]. From these observations, high-risk perception for HBV can translate to high vaccination coverage and completion of the recommended 3-dose regimen. Therefore interventions to improve the uptake of three-dose HBV vaccination should target activities that can improve HCWs’ risk perception of risk for HBV.

Participation in in-service training on the prevention of blood-borne infections was also significantly associated with the receipt of three doses of the HBV vaccine. This finding is similar to what was found in Ethiopia which suggests that attending training workshops on the prevention of blood-borne infections increases the odds of complete vaccination against HBV [28]. An in-service program is a professional training or staff development effort, where professionals are trained. This form of training is a key component of continuing medical education for HCWs and therefore is meant to improve the knowledge, practice, and performance of HCWs regarding patient care and their safety and protection [37,38]. Hence, it is not surprising that receipt of such training increased the odds of adherence to the 3-dose HBV vaccination regimen. On the contrary, a study among HCWs in Ghana that reported widespread in-service training for HCWs reported inadequate knowledge in HBV prevention [39] an indication of the poor impact of in-service training on health literacy and positive health behaviour among HCWs.

Seemingly, the results of the current study show that a high proportion (80.6%) of healthcare workers participated in workshops on the prevention and control of blood-borne infections. It is therefore unclear why less than 50% of them complied with the three-dose regimen, given the fact that receipt of in-service training improves adherence to the receipt of 3-doses of the HBV vaccine. Thus, apart from receiving training in blood-borne infection prevention, other structural barriers within the healthcare environment could have contributed to the failure to adhere to the 3-dose regimen. These structural barriers may include the unavailability of HBV vaccines in the facility, the absence of occupational health and safety programs to drive HCWs vaccination programs [40], lack of commitment and support from facility management, and poor coordination of infection prevention programs [41].

### Recommendations based on findings

Based on the findings of this study, we recommend compulsory, quality, and periodic in-service training sessions on the prevention of blood-borne infections for HCWs at all levels of care in the healthcare delivery system. Mandatory vaccination programs by health institutions can improve vaccination uptake in general and adherence to the three-dose HBV vaccination protocol. In as much as the findings of this present study emphasize the need to improve the quality and frequency of in-service training programs, there is also an urgent need to remove the structural or institutional barriers that might affect adherence to HBV vaccination protocol.

### Study limitations

The study being a cross-sectional study could not ascertain the exact cause of non-adherence to the recommended three doses of the HBV vaccine. A few participants (1.8%) did not have evidence of receipt of the 3 doses of the vaccine. In these few instances, self-reports were utilized. This is a limitation however the proportion based on self-report (1.8%) is too small to have any significant effect on factors associated with receipt of three doses of HBV vaccine estimated in this study.

The design of the study did not allow the researchers to assess the impact of vaccine safety, previous side effects, knowledge, attitude/belief on vaccines, normative beliefs, subjective norms, and peer/ Social influences on the completion of the three-dose cycle of the vaccine. And therefore, we consider this as a limitation to our study.

### Conclusion

Uptake of the complete cycle of three doses of HBV vaccine is not universal among the health care workers in the Greater Accra Region of Ghana. Good risk perception for HBV infection and training in infection prevention were statistically associated with the receipt of three doses of the vaccine. Interventions to increase risk perception through workshops and training in the prevention of blood-borne infections could help improve adherence to the complete cycle of the dose vaccination protocol. This study is important because it has highlighted and therefore identified HBV vaccination practices of HCWs in the Greater Accra Region. To improve HCWs’ safety and reduce the risk of the occupational acquisition of HBV, there is an urgent need to remove structural and institutional barriers that influence the receipt of the three doses HBV vaccine.

## Supporting information

S1 File Vaccination data. (XLSX)
